# Supplementary figures and images for: TcpC inhibits toll-like receptor signaling pathway by serving as an E3 ubiquitin ligase that promotes degradation of myeloid differentiation factor 88
Source: PLoS Pathog. 2021 Mar 31;17(3):e1009481. doi: 10.1371/journal.ppat.1009481 (PMC8041205; doi:10.1371/journal.ppat.1009481)

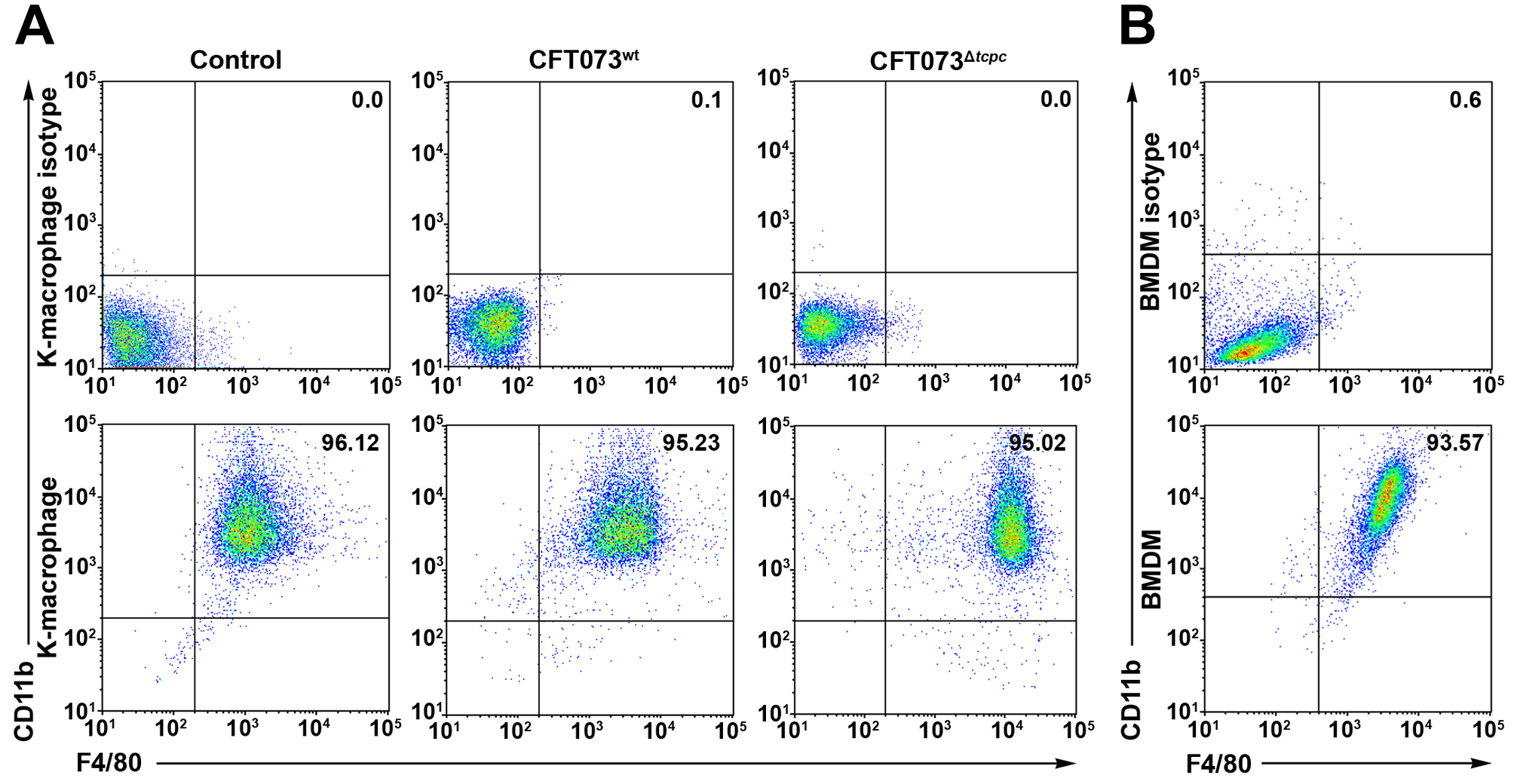

Supplement: S1 Fig — (A) CD11b and F4/80 expressions in K-macrophages isolated from control and PN mice models. (B) CD11b and F4/80 expressions in BMDM. (TIF) [file ppat.1009481.s001.tif]

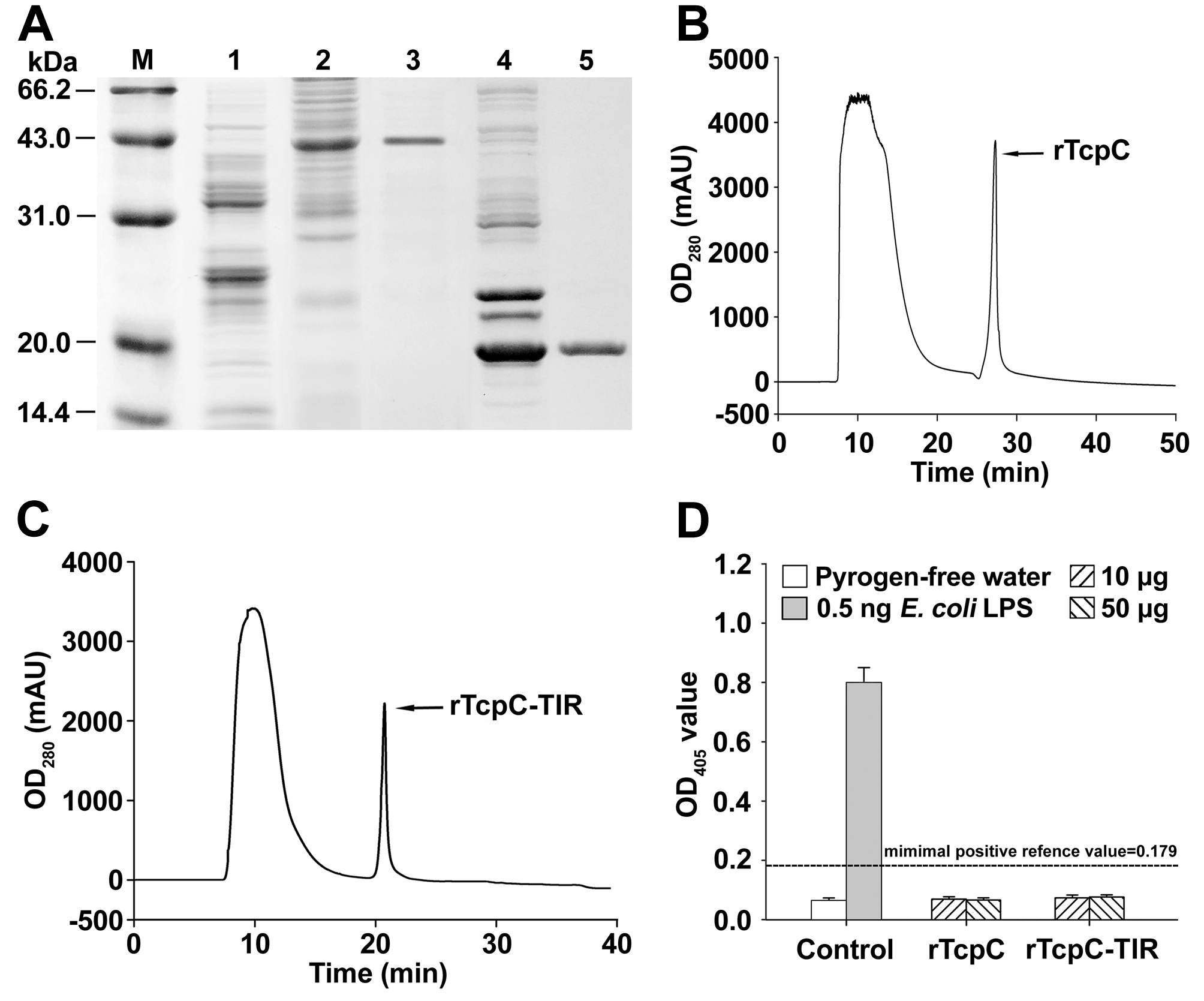

Supplement: S2 Fig — (A) Expression and purification of rTcpC and rTcpC-TIR. Lane M: protein marker. Lane 1: pET42a transformed E. coli BL21DE3. Lane 2: pET42a-tcpc transformed E. coli strain BL21DE3. Lane 3: Purified rTcpC. Lane 4: pET42a-tcpc-tir transformed E. coli strain BL21DE3. Lane 5: Purified rTcpC-TIR. (B) Elution curve of rTcpC. (C) Elution curve of rTcpC-TIR. (D) Detection of LPS in rTcpC or rTcpC-TIR preparation. (TIF) [file ppat.1009481.s002.tif]

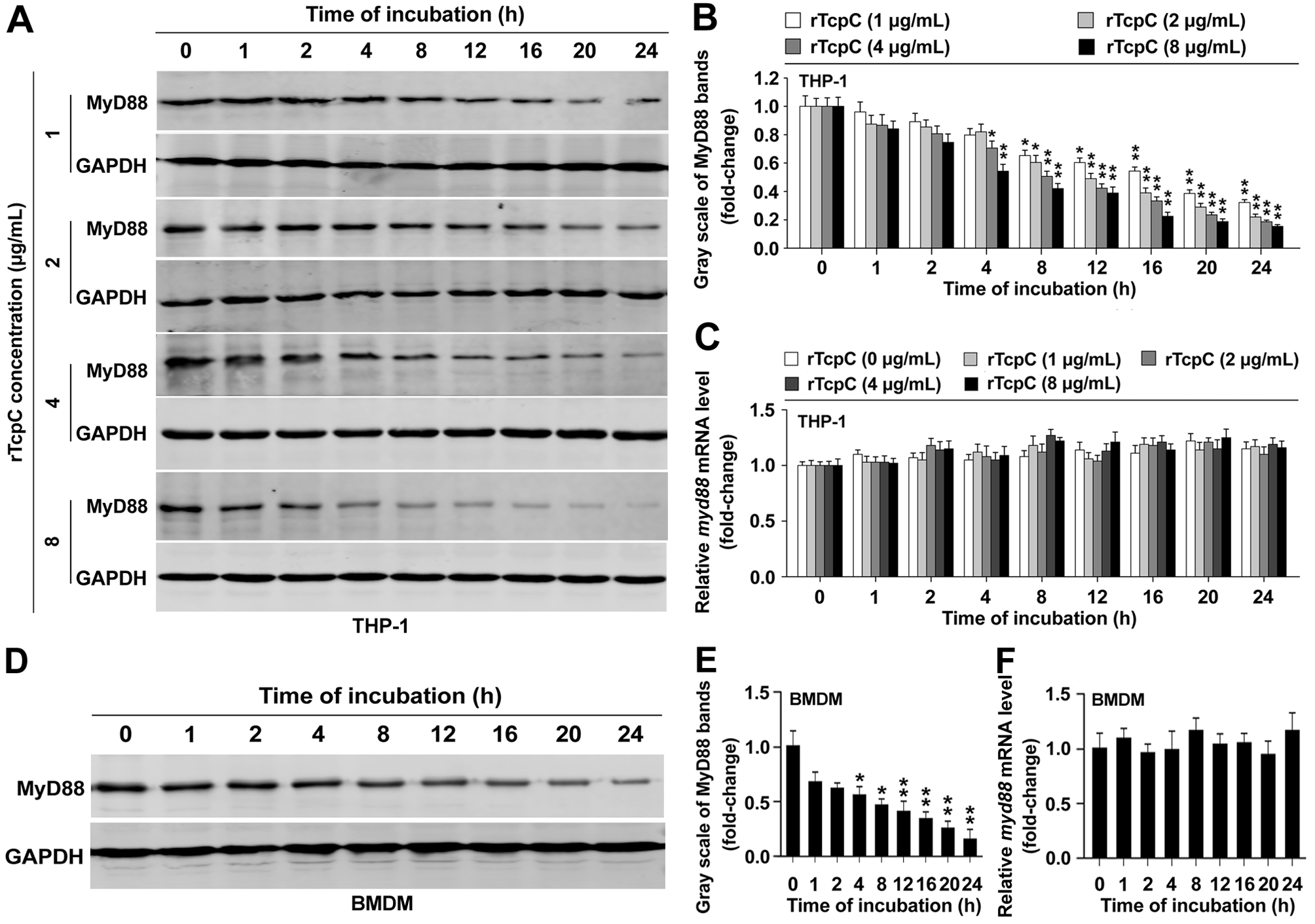

Supplement: S3 Fig — (A) Dose-dependent inhibitory effects of rTcpC on MyD88 protein levels in THP-1. (B) Gray scale analyses of MyD88 bands in THP-1 treated with different doses of rTcpC. Mean ± SD of three independent experiments were shown. The MyD88 protein levels in cells without rTcpC treatment were set as 1.0. *: p<0.05, **: p<0.01 vs the gray scale values reflecting the MyD88 levels in cells without rTcpC treatment. (C) Dynamic analyses, by qRT-PCR, of the influence of rTcpC on myd88 mRNA levels in THP-1. Mean ± SD of three independent experiments were shown. The myd88 mRNA levels in cells without rTcpC treatment were set as 1.0. (D) Inhibitory effects of rTcpC on MyD88 protein levels in BMDM. (E) Gray scale analyses of MyD88 bands in experiments as described in D. Mean ± SD of three independent experiments were shown. The MyD88 protein levels in cells without rTcpC treatment were set as 1.0. *: p<0.05, **: p<0.01 vs the gray scale values reflecting the MyD88 levels in cells without rTcpC treatment. (F) Dynamic analyses of the influence of rTcpC on myd88 mRNA levels in BMDM. (TIF) [file ppat.1009481.s003.tif]

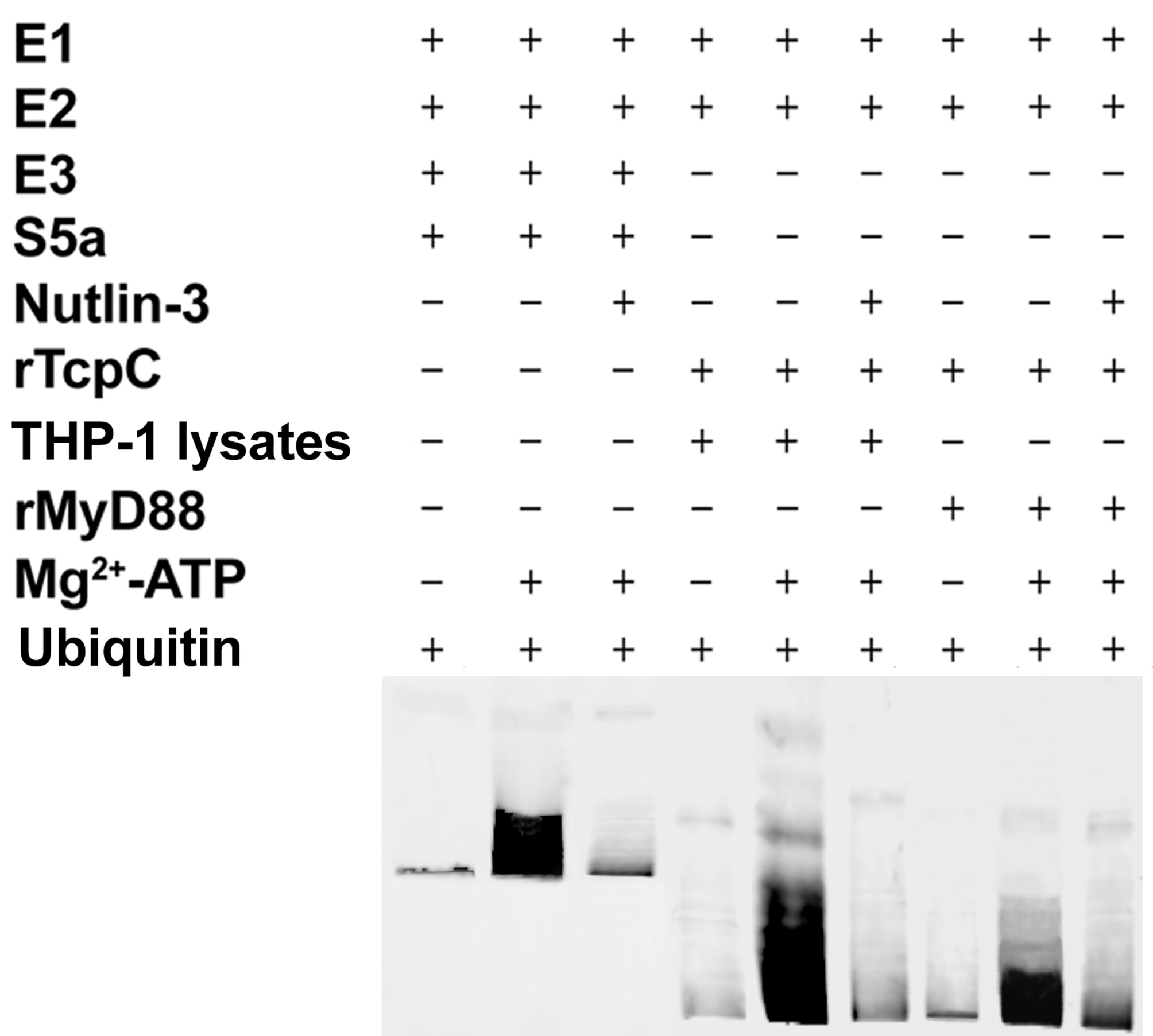

Supplement: S4 Fig — Ubiquitination kit tests to detect the E3 activity of rTcpC. S5a, an E3 ubiquitin ligase enzyme substrate was used as the control. rTcpC was used as the E3 when lysates from THP-1 and rMyD88 used as the substrates. (TIF) [file ppat.1009481.s004.tif]

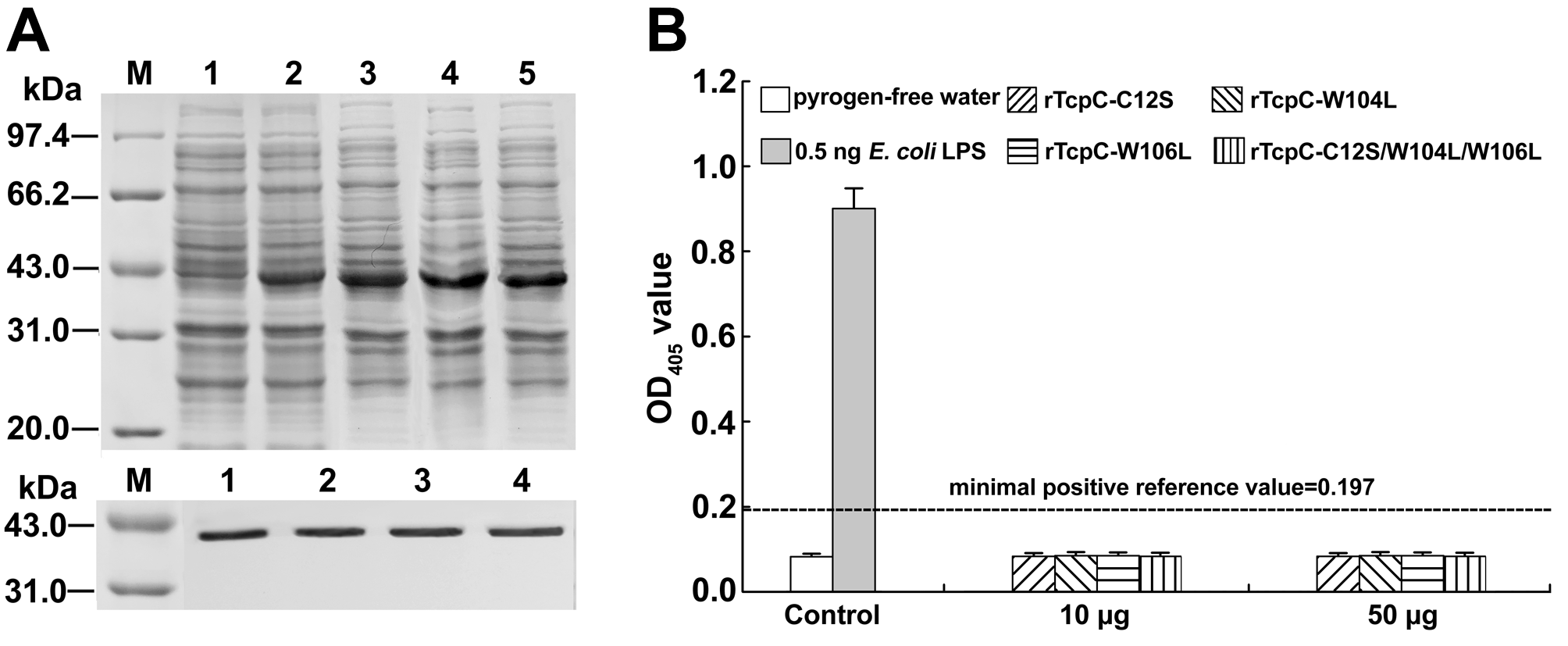

Supplement: S5 Fig — (A) SDS-PAGE analyses of expression and purification of rTcpC mutants. Upper: Expression of rTcpC mutants. Lane M: Protein marker. Lane 1: pET42a transformed E. coli BL21DE3. Lanes 2–5: pET42a-tcpc-C12S-, pET42a-tcpc-W104L-, pET42a-tcpc-W106L- and pET42a-tcpc-C12S/W104L/W106L-transformed E. coli BL21DE3, respectively. Bottom: Purity identification of rTcpC mutants. Lane M: Protein marker. Lane 1–4: rTcpC-C12S, rTcpC-W104L, rTcpC-W106L and rTcpC-C12S/W104L/W106L, respectively. (B) Detection of LPS in rTcpC mutants by spectrophotometric limulus test. (TIF) [file ppat.1009481.s005.tif]

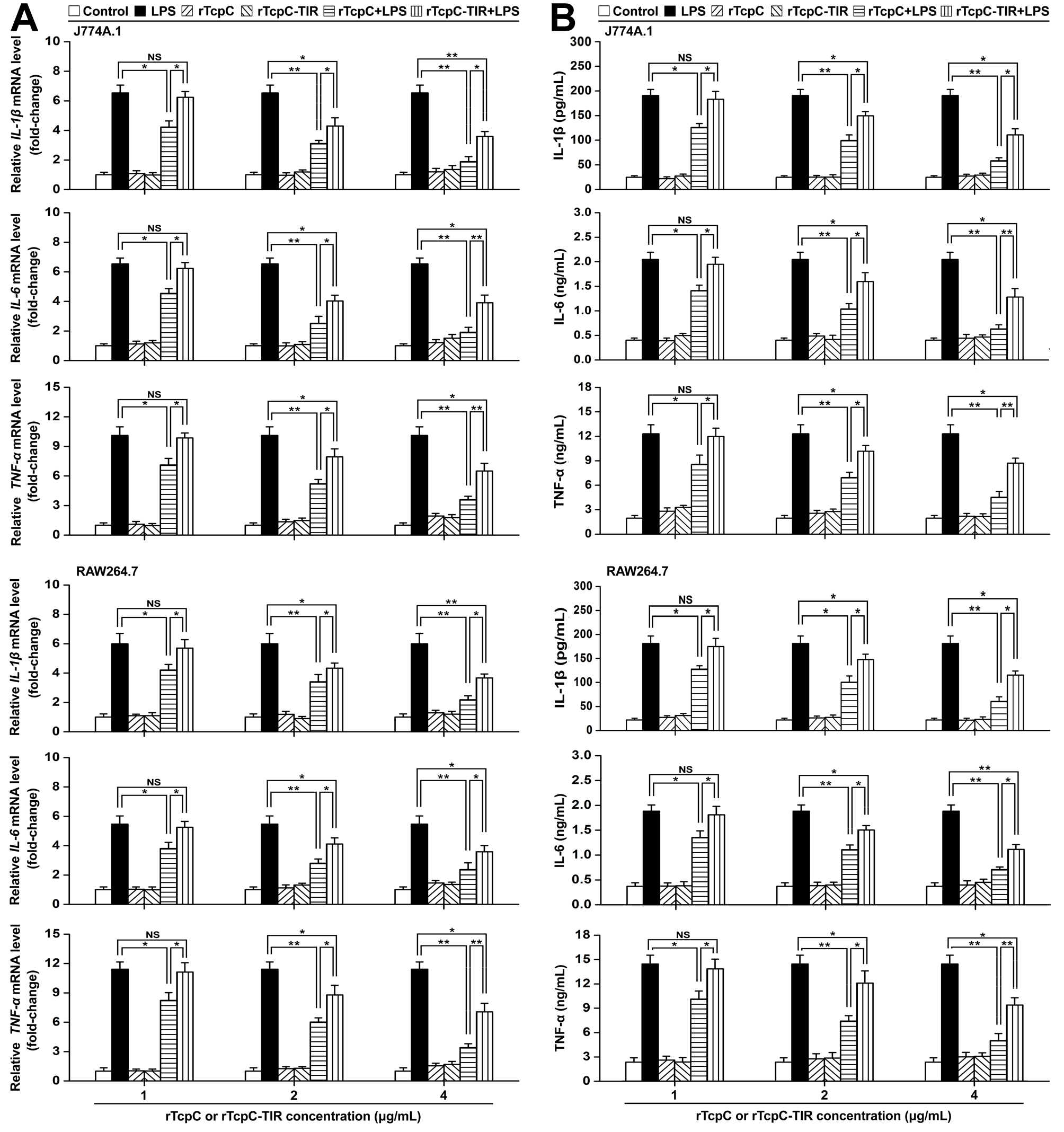

Supplement: S6 Fig — (A) qRT-PCR to examine the influence of rTcpC and rTcpC-TIR on mRNA levels of IL-1β, IL-6 and TNF-α. *: p<0.05, **: p<0.01, NS: no sense. (B) ELISA to detect protein levels of IL-1β, IL-6 and TNF-α. *: p<0.05, **: p<0.01, NS: no sense. (TIF) [file ppat.1009481.s006.tif]
