# Supplementary material for: TcpC inhibits toll-like receptor signaling pathway by serving as an E3 ubiquitin ligase that promotes degradation of myeloid differentiation factor 88
Source: PLoS Pathog. 2021 Mar 31;17(3):e1009481. doi: 10.1371/journal.ppat.1009481 (PMC8041205; doi:10.1371/journal.ppat.1009481)
Supplement: S1 Table — (DOCX) [file ppat.1009481.s007.docx]

**S1 Table. Primers used in** **this study**

| Primer | Sequence (5’ to 3’) | |
| --- | --- | --- |
| *tcpc*  *tcpc-tir*  *tcpc1* | | F: CGGATCCATGATAGCATATGAA  R: GGGAATTCTCTTCTCCTGTATGC  F: CGCCATATGCACTATGATTTTTTCATATCC  R: CGCCTCGAGTCTTCTCCTGTATGCTATTTC  F: TTTATA**AGC**TTGGTGAATGTTTTGGGC |
|  | | R: TCACCAAGCTTATAAAAAATTCTATGTTTTCATATGCTATCAC |
| *tcpc2* | | F: ATAGA**TTG**GAGTGGAAGGAGGTTG |
|  | | R: CCACTCCAATCTATAATCGTGGATATAAGAAATTGAT |
| *tcpc3* | | F: GGGAG**TTG**AAGGAGGTTGAGGCAAAG  R: CCTTCAACTCCCATCTATAATCGTGGA |

F: forward primer. R: reverse primer. *tcpc1*: *tcpc* with C12S point mutation; *tcpc2*: *tcpc* with W104L point mutation; *tcpc3*: *tcpc* with W106L point mutation.
