# Supplementary material for: TcpC inhibits toll-like receptor signaling pathway by serving as an E3 ubiquitin ligase that promotes degradation of myeloid differentiation factor 88
Source: PLoS Pathog. 2021 Mar 31;17(3):e1009481. doi: 10.1371/journal.ppat.1009481 (PMC8041205; doi:10.1371/journal.ppat.1009481)
Supplement: Attachment — Submitted filename: Rev2.pdf [file ppat.1009481.s011.pdf]

PPATHOGENS-D-20-02098

TcpC inhibits toll-like receptor signaling pathway by serving as an E3 ubiquitin ligase that promotes degradation of MyD88 by Jian-ping Pan et al.

This study investigates the bacterial protein TcpC and its role as a virulence factor of uropathogenic *E. coli* (UPEC). Previous studies have shown that the TIR domain of TcpC inhibits TLR signaling by direct association with MyD88. Here, a different approach is taken, as the authors investigate whether bacterial pathogens further manipulate the MyD88-dependent response by regulating MyD88 levels in host cells by stimulating MYC degradation. The authors propose that TcpC is a MyD88-targeted E3 ubiquitin ligase. Decreased MyD88 levels were detected in kidney macrophages from mice with acute pyelonephritis, when infected with TcpC-secreting CFT073 bacteria, but not a TcpC deletion mutant of this strain. Recombinant TcpC reduced MyD88 protein levels but did not affect gene expression, suggesting a protein effect. A mechanism of MYC degradation was suggested as rTcpC promoted MyD88 ubiquitination and proteasome accumulation in macrophages. The results suggest that TcpC reduces cellular MyD88 levels by ubiquitination and increased proteasomal degradation, thereby inhibiting TLR signaling.

These are interesting observations and the data is mostly convincing. A few questions remain to be addressed, however.

1. The presentation of the infection data is somewhat superficial. It would be useful if the photographs of infected kidneys and inflammatory cell infiltrates were accompanied by data indicating group numbers and statistics in the same figure. How many mice in each group developed macroscopic signs of acute pyelonephritis? Did the authors construct a pathology score weighing the numbers of inflammatory cells and effects on tissue integrity? Which inflammatory cells?

2. The rationale for the LAMP1 staining analysis in Fig. 1 is not clear and the difference in MYD88 levels is not impressive.

3. The effect of rTcpC on cytokine responses is convincing, as is the effect on bacterial survival in macrophages.

4. The co-IP and analysis of proteins in cell lysates is a bit unclear. A direct interaction of rTcpC with MyD88 would be expected, based on the known TIR domain recognition mechanism. The precipitation of ubiquitinated MyD88 needs further clarification.

5. The authors show that rTcpC can replace ubiquitin ligase and elegantly trace the peptide-specific ubiquitination sites. The ubiquitination reactions are detected using a kit and cell lysates as expected from the need for cellular cofactors to support these reactions. How was the experiment designed to detect the ubiquitination of rMyD88?

6. The LAMP1 staining and co-localization is clear but it might be of interest to examine the cells further for interactions with specific proteasome constituents. If Fig. 1 is extended to include more *in vivo* data, the Lamp1 staining could be moved to the later Figure.
