# Supplementary material for: TcpC inhibits toll-like receptor signaling pathway by serving as an E3 ubiquitin ligase that promotes degradation of myeloid differentiation factor 88
Source: PLoS Pathog. 2021 Mar 31;17(3):e1009481. doi: 10.1371/journal.ppat.1009481 (PMC8041205; doi:10.1371/journal.ppat.1009481)
Supplement: Attachment — Submitted filename: PPATHOGENS-D-20-02098_reviewer to submit.docx [file ppat.1009481.s010.docx]

Urinary tract infection is a widely common recurrent infection and Uropathogenic *E. coli* (UPEC) are responsible for about 80% of infections in the world. In this study the authors show that a virulence factor from UPEC, called TcpC, works as a Myd88-target E3 ubiquitin ligase in host cells. By using a wild-type and mutant UPEC lacking TcpC, the authors have shown that Myd88 protein levels are decreased in macrophages from mice infected with wild-type UPEC, while co-localization with the proteasome in the cell was enhanced compared to cells from mice infected with UPEC lacking TcpC. In addition, macrophage treatment with recombinant TcpC promoted Myd88 ubiquitination and accumulation in the proteasome. Previous reports have shown that TcpC impedes Toll like receptor activation by direct competitive binding to Myd88. It is very interesting that TcpC can also work as a Myd88 ubiquitin E3 ligase to suppress the inflammatory response in host cells during infection. However, the work is missing correct explanation of controls used or proper controls, a more detailed description of experiments performed, and correct discussion of the study. Concerns and questions are as follow:

1. In figure 1, authors use 3 conditions, which includes control group, mice infected with wt UPEC, and mice infected with mutant UPEC. What is the control group? Naïve mice? Also

in figure 2, authors again do not explain what is the control group used. In figure 2A and 2B, the control group shows living bacteria. However, what exactly is this control group? Are mice infected with another bacterial strain? Which one? What is staining green if there are no bacteria present?

2. Related to figure 2A, the experiment was performed by infecting macrophages *in vitro* and washing several times with PBS to obtain cell pellets and quantify intracellular bacteria. Using this approach, it is not possible to confidently quantify intracellular bacteria, because bacteria that are adherent on the surface of macrophages can be erroneously counted as intracellular. The authors should perform an antibiotic/gentamicin protection assay to be sure they are killing all extracellular bacteria.
3. The results section for figure 3 (lines 142 to 144) and figure 4 (lines 185 to 187) would be more clear and easier to understand if the authors gave a more detailed description of the experimental approach, instead of directly stating the results obtained.
4. The authors suggest that MyD88 protein levels are being degraded due to TcpC mediated ubiquitination. However, is it possible, and not mutally exclusive, that MyD88 protein levels are also reduced over time due to reduced mRNA translation? Can the authors test or discuss that, or rule out the possibility?
5. In Figure 4A and 4B, authors say on line 158 “ubiquitination of MyD88 was enhanced profoundly compared with the control group”. What is the control group the authors are referring about? Also, is it possible to quantify the bands to do statistics between groups?
6. The authors did lot of immuno blot analysis, but the raw data blots are not provided with the protein weight/ladder for review.
7. Overall, the statistics used are not appropriate –ANOVA assume the data are distributed normally. Given the small numbers, it is not possible to determine whether these data are normally distributed – and as such, nonparamentric Mann-Whitney or Kruskal-Wallis tests should be used – and in the case of a Kruskal-Wallis, a post-test should be used and the type of post-test should be stated in the methods. If data were tested for normality, or transformed to obtain normality before ANOVA test, it should be described in methods section. It is generally difficult to test for normality, however, in groups with a small n (<30), so it is better to use a non-parametric test that does not assume normality.
8. In the discussion, the authors do not discuss their data in the context of the published literature. The discussion looks like another description of the results section. From line 236 to line 268, the authors only describe the TLR signaling pathway. This could be cut to 2-3 lines, or even cut entirely. Then, from line 269 to line 355 (3 pages), authors just extensively describe their results section again. What are the implications of this work? How do the authors explain their findings in light of that of previously published work on TcpC? Are there other bacterial virulent factors, especially UPEC virulent factors, that behave the same? How could this impact the immune response to UPEC? These and many other questions could be discussed in this section.
9. It is highly unusual to have a supplementary results and supplementary methods in addition to supplementary figures and legends. Why are these results not integrated into the text?
