# Supplementary material for: TcpC inhibits toll-like receptor signaling pathway by serving as an E3 ubiquitin ligase that promotes degradation of myeloid differentiation factor 88
Source: PLoS Pathog. 2021 Mar 31;17(3):e1009481. doi: 10.1371/journal.ppat.1009481 (PMC8041205; doi:10.1371/journal.ppat.1009481)
Supplement: Attachment — Submitted filename: Rebuttal letter to comments of the reviewers.docx [file ppat.1009481.s012.docx]

**Prof. Dr. Jianping Pan**

Institute of Translational Medicine

Zhejiang University City College

50 Huzhou Road, Hangzhou 310015

P. R. China

Tel: +86 571 88285702

Fax: +86 571 88018442

E-Mail: jppan@zucc.edu.cn

Jan. 20, 2021

**Re.: Manuscript PPATHOGENS-D-20-02098 by Fang et al: Revised Version**

Dear Editor,

We have revised, according to the comments of the reviewers, our manuscript entitled “TcpC inhibits toll-like receptor signaling pathway by serving as an E3 ubiquitin ligase that promotes degradation of myeloid differentiation factor 88” (PPATHOGENS-D-20-02098).

We have included all the additional requested experiments in the revised manuscript: 1) the viability of UPEC from infected macrophages with perform an antibiotic protection assay to killing all extracellular bacteria; 2) the influence of rTcpC on the protein level of MyD88 in human macrophage cell line THP-1 and mouse BMDM; 3) the influence of rTcpC on the mRNA level of *myd88* in THP-1 and BMDM; 4) the influence of rTcpC on the ubiquitination level of MyD88 or rMyD88 in THP-1.

We have also corrected some spelling errors in the text that were not mentioned by the reviewers and the format of manuscript.

As you will see from the revised manuscript, we have performed additional experiments and revisions to address the issues raised by the reviewers, and we believe that we have adequately addressed their criticism. Therefore, we would be pleased if the revised manuscript would now be accepted for publication in “PLoS Pathogens”.

Please find the point-by-point responses to the reviewers’ comments enclosed.

Sincerely yours,

Jianping Pan

**Reviewer #1:**

Urinary tract infection is a widely common recurrent infection and Uropathogenic *E. coli* (UPEC) are responsible for about 80% of infections in the world. In this study the authors show that a virulence factor from UPEC, called TcpC, works as a Myd88-target E3 ubiquitin ligase in host cells. By using a wild-type and mutant UPEC lacking TcpC, the authors have shown that Myd88 protein levels are decreased in macrophages from mice infected with wild-type UPEC, while co-localization with the proteasome in the cell was enhanced compared to cells from mice infected with UPEC lacking TcpC. In addition, macrophage treatment with recombinant TcpC promoted Myd88 ubiquitination and accumulation in the proteasome. Previous reports have shown that TcpC impedes Toll like receptor activation by direct competitive binding to Myd88. It is very interesting that TcpC can also work as a Myd88 ubiquitin E3 ligase to suppress the inflammatory response in host cells during infection. However, the work is missing correct explanation of controls used or proper controls, a more detailed description of experiments performed, and correct discussion of the study.

**Reply:** We thank the reviewer for a thorough review of our manuscript and for providing excellent comments for improvement. We have now addressed all concerns raised and made substantial improvements in the revised manuscript.

1. In figure 1, authors use 3 conditions, which includes control group, mice infected with wt UPEC, and mice infected with mutant UPEC. What is the control group? Naïve mice? Also in figure 2, authors again do not explain what is the control group used. In figure 2A and 2B, the control group shows living bacteria. However, what exactly is this control group? Are mice infected with another bacterial strain? Which one? What is staining green if there are no bacteria present?

**Reply:** In figure 1, wt UPEC and mutant UPEC were grown in LB medium and harvested by centrifugation at 12,000×g for 20 min and resuspended in normal saline to a final concentration of 1×10^9^. Therefore, normal saline-treated mice served as the control group, which was described in“ Mouse pyelonephritis model” on page 17, line 378. In figure 2A and 2B, wt UPEC or mutant UPEC cultured in LB medium were used as the control groups that was now labeled in figure 2 directly.

2. Related to figure 2A, the experiment was performed by infecting macrophages *in vitro* and washing several times with PBS to obtain cell pellets and quantify intracellular bacteria. Using this approach, it is not possible to confidently quantify intracellular bacteria, because bacteria that are adherent on the surface of macrophages can be erroneously counted as intracellular. The authors should perform an antibiotic/gentamicin protection assay to be sure they are killing all extracellular bacteria.

**Reply:** According to the criticism raised by the reviewer, we have performed an antibiotic protection assay to be sure that the extracellular attached bacteria are killed before washing several times with PBS to obtain cell pellets and quantify intracellular bacteria and provide the new figure 2A. We also revised the experiment method in Materials and methods section on page 19, lines 420-421 in the revised manuscript.

3. The results section for figure 3 (lines 142 to 144) and figure 4 (lines 185 to 187) would be more clear and easier to understand if the authors gave a more detailed description of the experimental approach, instead of directly stating the results obtained.

**Reply:** We have provided detailed description of the experiments in figure 3 in the section of Materials and methods on page 18, lines 387-393 and added the description of experimental approach in figure 4 in figure legend on page 33, lines 729-733.

4. The authors suggest that MyD88 protein levels are being degraded due to TcpC mediated ubiquitination. However, is it possible, and not mutally exclusive, that MyD88 protein levels are also reduced over time due to reduced mRNA translation? Can the authors test or discuss that, or rule out the possibility?

**Reply:** To detect the influence of rTcpC on mRNA expression of *myd88*, J774A.1, RAW264.7, THP-1 were treated with different concentrations of rTcpC (1, 2, 4 or 8 μg/ml), and BMDM were treated with 4 μg/ml rTcpC for 1, 2, 4, 8, 12, 16, 20 or 24 h respectively. Dynamic analyses of rTcpC on the mRNA levels of *myd*88 were examined by qRT-PCR. Our data showed that rTcpC did not influence *myd88* mRNA levels in macrophages during the whole treatment period of time (Figure 3E and 3F and S3C and S3F Figure). Yes, the reviewer is right, it is possible that the decreased protein levels of MyD88 in rTcpC treated macrophages are due to the reduced mRNA translation over time. But our data showed that TcpC treatment resulted in enhanced ubiquitination of MyD88 (Fig 4 and S4 Fig) and promoted cololization of MyD88 with proteasome (Fig 1G and Fig 6), which strongly support our conclusion that TcpC promote degradation of MyD88.

5. In Figure 4A and 4B, authors say on line 158 “ubiquitination of MyD88 was enhanced profoundly compared with the control group”. What is the control group the authors are referring about? Also, is it possible to quantify the bands to do statistics between groups?

**Reply:** In Figure 4A and 4B, control group refers to rTcpC untreated, we made explanation of the control group in Results section on page 8, line 170 in the revised manuscript. According to the reviewer’s request, we have quantified the bands to do statistics analyses between groups, which was shown in Figure 4C and 4D.

6. The authors did lot of immuno blot analysis, but the raw data blots are not provided with the protein weight/ladder for review.

**Reply:** We will provide the raw data of immuno blot bands when resubmit the revised manuscript for review.

7. Overall, the statistics used are not appropriate –ANOVA assume the data are distributed normally. Given the small numbers, it is not possible to determine whether these data are normally distributed – and as such, nonparamentric Mann-Whitney or Kruskal-Wallis tests should be used – and in the case of a Kruskal-Wallis, a post-test should be used and the type of post-test should be stated in the methods. If data were tested for normality, or transformed to obtain normality before ANOVA test, it should be described in methods section. It is generally difficult to test for normality, however, in groups with a small n (<30), so it is better to use a non-parametric test that does not assume normality.

**Reply:** The reviewer is right. We have used non-parametric Mann-Whitney test to replace ANOVA test in Materials and methods section on page 24, line 516 in the revised manuscript.

8. In the discussion, the authors do not discuss their data in the context of the published literature. The discussion looks like another description of the results section. From line 236 to line 268, the authors only describe the TLR signaling pathway. This could be cut to 2-3 lines, or even cut entirely. Then, from line 269 to line 355 (3 pages), authors just extensively describe their results section again. What are the implications of this work? How do the authors explain their findings in light of that of previously published work on TcpC? Are there other bacterial virulent factors, especially UPEC virulent factors, that behave the same? How could this impact the immune response to UPEC? These and many other questions could be discussed in this section.

**Reply:** According to the reviewer’s request, we have revised the Discussion section and pointed out that a number of bacterial pathogen derived E3 have been found, but none of them are E3 that promote ubiquination of proteins in the TLR signalling pathway, on page 15, lines 333-338 in the revised manuscript. We have also pointed out the significance of our findings in the last paragraph of the discussion section.

9. It is highly unusual to have a supplementary results and supplementary methods in addition to supplementary figures and legends. Why are these results not integrated into the text?

**Reply:** We have integrated supporting results and supporting methods into the text in the revised manuscript.

**Reviewer #2:**

This study investigates the bacterial protein TcpC and its role as a virulence factor of uropathogenic *E. coli* (UPEC). Previous studies have shown that the TIR domain of TcpC inhibits TLR signaling by direct association with MyD88. Here, a different approach is taken, as the authors investigate whether bacterial pathogens further manipulate the MyD88-dependent response by regulating MyD88 levels in host cells by stimulating MYC degradation. The authors propose that TcpC is a MyD88-targeted E3 ubiquitin ligase. Decreased MyD88 levels were detected in kidney macrophages from mice with acute pyelonephritis, when infected with TcpC-secreting CFT073 bacteria, but not a TcpC deletion mutant of this strain. Recombinant TcpC reducedMyD88 protein levels but did not affect gene expression, suggesting a protein effect. A mechanism of MYC degradation was suggested as rTcpC promoted MyD88 ubiquitination and proteasome accumulation in macrophages. The results suggest that TcpC reduces cellular MyD88 levels by ubiquitination and increased proteasomal degradation, thereby inhibiting TLR signaling. These are interesting observations and the data is mostly convincing. A few questions remain to be addressed, however.

1. The presentation of the infection data is somewhat superficial. It would be useful if the photographs of infected kidneys and inflammatory cell infiltrates were accompanied by data indicating group numbers and statistics in the same figure. How many mice in each group developed macroscopic signs of acute pyelonephritis? Did the authors construct a pathology score weighing the numbers of inflammatory cells and effects on tissue integrity? Which inflammatory cells?

**Reply:** The reviewer’s opinion is reasonable. 7 of 9 mice developed macroscopic signs of acute pyelonephritis in CFT073^wt^ group but none in other groups. We made a table in figure 1B and presented the photographs of HE staining and statistical analysis of neutrophil infiltration for the histosection of kidney in figure 1C and 1D. And provide detailed description of the experiments in Materials and methods section on page 17, lines 375-379, and added the related information in figure leged on page 31, lines 674-678 in the revised manuscript.

2. The rationale for the LAMP1 staining analysis in Fig. 1 is not clear and the difference in MYD88 levels is not impressive.

**Reply:** The process of protein degradation by ubiquitin-proteasome system is an activating enzyme E1, transfers ubiquitin to a carrier E2 enzyme, which in turn tags ubiquitin to the doomed substrate with the help of E3 enzymes, delivery and accumulate to 26S proteasomes finally [1, 2]. The level of accumulation for MyD88 was enhanced profoundly, while co-localization of MyD88 with the proteasome marker LAMP1 was enhanced significantly in kidney macrophages (K-macrophages) from CFT073^wt^ infected mice with pyelonephritis compared to K-macrophages isolated from mice infected with CFT073^Δ^*^tcpc^* (Figure 1G-1I). Therefore, these data suggest that TcpC might promote degradation of MyD88.

[1] Pohl C, Dikic I. Cellular quality control by the ubiquitin-proteasome system and autophagy. Science 2019; 366(6467): 818-822.

[2] Collins GA, Goldberg AL. The logic of the 26S proteasome. Cell 2017; 169(5): 792-806.

3. The effect of rTcpC on cytokine responses is convincing, as is the effect on bacterial survival in macrophages.

**Reply:** We thank the reviewer for a thorough review of effect of rTcpC on cytokine responses and bacterial survival in macrophages.

4. The co-IP and analysis of proteins in cell lysatesis a bit unclear. A direct interaction of rTcpC with MyD88 would be expected, based on the known TIR domain recognition mechanism. The precipitation of ubiquitinated MyD88 needs further clarification.

**Reply:** In order to explain ubiquitinated MyD88 more intuitively, we use software to quantify the bands to do statistics between groups in Figure 4C and 4D.

5. The authors show that rTcpC can replace ubiquitin ligase and elegantly trace the peptide-specific ubiquitination sites. The ubiquitination reactions are detected using a kit and cell lysates as expected from the need for cellular cofactors to support these reactions. How was the experiment designed to detect the ubiquitination of rMyD88?

**Reply:** We use rTcpC or commercially available recombinant MyD88 (rMyD88) replace the E3 ubiquitin ligase or substrate protein for the ubiquitination kit tests, and results demonstrated that rTcpC could replace the E3 ubiquitin ligase for strengthening ubiquitination of rMyD88. Furthermore, this enhanced ubiquitination could be abrogated by the E3 ubiquitin ligase inhibitor Nutlin-3 (Fig 4I and 4J). In fact, the cell lysates only provide the substrate protein of the E3, E1, E2 and other cofactors, such as ATP, ubiquitin, are added in vitro as performed according to the instruction of the manufactureer.

6. The LAMP1 staining and co-localization is clear but it might be of interest to examine the cells further for interactions with specific proteasome constituents. If Fig.1 is extended to include more *in vivo* data, the Lamp1 staining could be moved to the later Figure.

**Reply:** The reviewer’s opinion is reasonable. But LAMP1 is one of the generally accepted markers of proteasome, therfore we use this marker to examine the colocalization of MyD88 with proteasome. We think that colocalization of MyD88 with proteasome is an important evidence to support our postulation that TcpC might promote degradation of MyD88, therefore we still put this figure as a part of Figure 1.

**Reviewer #3:**

Summary: The paper by Fang et al. presents interesting and novel data regarding the mechanism through which TcpC affects the host immune response via MyD88 protein.

The authors use a tcpC mutant strain compared to a wild-type strain to demonstrate the importance of the protein for virulence/colonization during UTI in a mouse model as well as entry/survival in macrophage and macrophage cell lines of murine origin. Globally, the study is well conducted and experiments well planned. The text is also clearly organized and written.

Strengths of the work include the combination of methods used to demonstrate the activity of the TcpC protein to act as a ubiquitin ligase and decrease levels of MyD88 protein as well as demonstration of key roles of specific residues for this activity, as well as demonstration of the partner proteins identified by mass spectrometry.

A drawback of the study concerns use of only mouse derived cells and protein although it is likely that this bacterial protein acts similarly on human host targets to abate the innate immune response.

**Reply:** We thank the reviewer for a thorough review of our manuscript and for providing excellent comments for improvement. Protein and mRNA expressions of MyD88 in rTcpC treated human macrophage cell line THP-1 cells were measured by Western blot and real-time qRT-PCR respectively (S3A-S3C Figure). Influence of rTcpC on the ubiquitination of THP-1 lysates or rMyD88 was also detected by ubiquitination kit (S4 Figure).

Major Issues:

For studies concerning macrophages or macrophage cell lines, all cell types tested are only murine source. Why were only mouse cell lines used? It would be worthwhile determining if the same responses occur with human derived macrophage cell lines or primary human macrophages. It would be of interest to confirm the same mechanism in a human source cell or cell line or human derived MyD88 protein sequence.

**Reply:** We have done the same experiments on human macrophage cell lin THP-1. rTcpC treatment dose dependently inhibits protein levels of MyD88 in THP-1, but does not affect its mRNA expression (S3A-S3C Figure). Ubiquitination kit tests demonstrated that rTcpC could enhance ubiquitination of THP-1 lysates or rMyD88. This enhanced ubiquitination of MyD88 by rTcpC could be abrogated by the E3 ubiquitin ligase inhibitor Nutlin-3 (S4 Figure).

Fig. 1. There seems to be a discoloration of the kidney with a paleness with WT CFT073, and an important increase in redness in the tcpC mutant infected kidneys. In B vs C is there a cellular content present that correlates with the change in color observed in the kideys? The term cellular infiltrates is vague and some of the basic changes in cell content could be described. More or less types of what cells??

**Reply:** Discoloration of the kidneys with a paleness of WT CFT073 was associated with severe abscesses. The increase of red color in the kidneys infected by tcpC mutant may be due to the slight inflammation caused by bacterial infection. We made a table in revised figure 1B and presented the photographs of HE staining and statistical analysis of neutrophil infiltration for the tissue section of kidney in revised figure 1C and 1D. We described some of the basic changes in neutrophil cells in Results section on page 6, line 119 in the revised manuscript.

Fig. 1C and D. The change in level of MyD88 detected seems to be potentially modest when considering differences in cellular composition in the tissues. This is an important aspect to consider from the wt vs. delta tcpC mutant infected kidneys and even the control samples (as seen in Fig. 1B). The enrichment used to obtain K-macrophages was cell sorting with the CD11b marker, which is not specific to macrophages, but common to most phagocytic and other cells (monocytes, neutrophils, macrophages, NK and even other cell types). To what extent can the differences in MyD88 quantification be attributed to cell composition versus differences in potential degradation or reduced levels of MyD88 in actual K-Macrophages when considering the Western blot results?

**Reply:** To maximally obtain K-macrophages, the cell suspension was allowed to incubate at 37°C for 2 h to let macrophages adhere to dish (while neutrophils and lymphocytes cannot) and the nonadherent cells were rinsed off with PBS before sorting. And the K-macrophage cell purity was >95% when identified by CD11b and F4/80 expression (S1 Figure). We provided detaied description of the experimental method in Materials and methods section in Supporting Information on page 1, lines 13-16.

Minor Issues:

Pathogenic *E. coli* causing extra-intestinal infections such as UTIs and sepsis represent a diverse group of isolates tat can contain a variety of virulence factors. This work focuses on a protein from a well studied from from a case of pyelonephritis. It would be worthwhile for the authors to also present a few sentences concerning the prevalence of TcpC among different ExPEC UPEC strains and among specific phylogenetic groups, sequence types, etc. There are quite a few publications describing the frequency of this virulence factor and its association with particular subgroups of *E. coli*. This would better substantiate the potential general role of TcpC for UTIs and other systemic infections cause by *E. coli*.

**Reply:** We thank the reviewer for a thorough review of our manuscript and for providing excellent comments for improvement. We present several sentences concerning the prevalence of TcpC among different ExPEC UPEC strains in Introduction section on page 4, lines 82-85 in the revised manuscript.

In Fig. 2a, it is unclear what the control shown is: the labeling indicates macrophages or cells lines, but is the control just bacteria that were not interacting with macrophage cells? It is also not clear why bacterial cell enumeration by viable counts after macrophage cell lysis was not determined as a direct method to quantify total differences in survival during macrophage interaction.

**Reply:** To more intuitively observe the influence of TcpC on the survival of UPEC in macrophages, confocal microscopy was employed using a LIVE/DEAD Bacterial Viability Kit. In Fig. 2A, CFT073^wt^ or CFT073^Δ^*^tcpc^* in LB medium were used as the control groups. We have performed an antibiotic protection assay to be sure that all the extracellular attached bacteria are killed before washing several times with PBS to obtain cell pellets and quantify intracellular bacteria and provide the new figure 2A. We improved experiment method in Materials and methods section on page 19, lines 420-421 in the revised manuscript.

Fig. 2. For plates G and H, the time of the sampling after infection should be indicated in the Figure legend.

**Reply:** We add the time of the sampling after infection in the Figure 2G and 2H legends.

Fig. 3. The names of the cell lines tested should be labeled directly in the figures for more clarity. It would have been of interest to determine if a similar decrease in MyD88 occured in primary macrophages as well.

**Reply:** We have added the names of the cell lines in Fig. 3. The reviewer’s opinion is reasonable. To detect the influence of rTcpC on the protein levels of MyD88, mouse bone marrow derived macrophages (BMDM) were treated with rTcpC (4 μg/ml) for the indicated time. Results mirrored the same trend were also obtained (S3D-S3F).

Fig. 7. Write the name of the cells as a label title above the Plates at top of Fig. 7, since J774 corresponds to A, C, E and RAW to B, D, F.

**Reply:** We have written the name of the cells in Fig. 7.

The discussion is limited in discussing the results in relation to other studies and is mainly presented as a revisitating of the data, making it rather repetitive. Without referring or comparing results or aspects to other studies, the discussion is lacking scope or comparison of other aspects related to discoveries presented, such as the existence of other bacterial ubiquitin ligases that play a role in virulence through different pathways for example. The paragraphs in the discussion are mainly repetitive descriptions of the results only, and need to be discussed in contrast or comparison to other reports or literature.

**Reply:** According to the reviewer’s request, we have revised the Discussion section and pointed out that a number of bacterial pathogen derived E3 have been found, but none of them are E3 that promote ubiquination of proteins in the TLR signalling pathway, on page 15, lines 333-338 in the revised manuscript. We have also pointed out the significance of our findings in the last paragraph of the discussion section.

Supplementary data:

Why are there no legends/text to explain what is shown in the plates in the supplemental figures? This should be independently clear without needing to refer to any text in other section or the main manuscript. Without legends, it is not possible to determine what is being presented.

**Reply:** According to the reviewer’s request, we explained the plates in the supplemental figures in Supporting Information in the revised manuscript.
