# Supplementary material for: TcpC inhibits toll-like receptor signaling pathway by serving as an E3 ubiquitin ligase that promotes degradation of myeloid differentiation factor 88
Source: PLoS Pathog. 2021 Mar 31;17(3):e1009481. doi: 10.1371/journal.ppat.1009481 (PMC8041205; doi:10.1371/journal.ppat.1009481)
Supplement: Attachment — Submitted filename: Rebuttal letter to comments.docx [file ppat.1009481.s013.docx]

**Prof. Dr. Jian-ping Pan**

Institute of Translational Medicine

Zhejiang University City College

50 Huzhou Road, Hangzhou 310015

P. R. China

Tel: +86 571 88285702

Fax: +86 571 88018442

E-Mail: jppan@zucc.edu.cn

Mar. 6, 2021

**Re.: Manuscript PPATHOGENS-D-20-02098R1 by Fang et al: Secondary revised version**

Dear Editor,

We have revised, according to the comments of the reviewers, our manuscript entitled “TcpC inhibits toll-like receptor signaling pathway by serving as an E3 ubiquitin ligase that promotes degradation of myeloid differentiation factor 88” (PPATHOGENS-D-20-02098R1).

We have made the following major revisions: 1) According to the suggestion of the reviewer, we used PSMD2 as the marker of proteasomes to more specifically verify the co-localization of MyD88 with proteasome in confocal microscopy; 2) We have revised the discussion section and pointed out the future research direction of the field.

As you will see from the secondary revised manuscript, we have performed additional experiments and revisions to address the issues raised by the reviewers, and we believe that we have adequately addressed their criticism. Therefore, we would be pleased if the manuscript would now be accepted for publication in “PLoS Pathogens”.

Please find the point-by-point responses to the reviewers’ comments enclosed.

Sincerely yours,

Jian-ping Pan

Reponses to reviewers

1. Previous reviewers questioned the use of LAMP1 as "one of the generally accepted markers of the proteasome". While LAMP1 is often used as a marker of lysosomes and occasionally autophagosomes, it is not clear that this is a commonly used proteasome marker. Please provide references for use of LAMP1 as a proteasome marker, or otherwise clarify this issue. For example, co-localization of MyD88 with proteasomes in a single cell line ± TcpC treatment using antibody specific for one of the proteasome subunits would be reassuring.

**Reply:** 26S proteasome is composed of 20S core particle and 19S regulatory particle^[1]^, and the Rpn1 or PSMD2 is a specific marker of the 19S subunit of proteasome ^[2,3]^. We re-examined the co-localization of MyD88 with PSMD2 instead of LAMP-1 by confocal microscopy. The data are now shown in Figure 1G-1I and Figure 6.

References:

[1] Bard JAM, Goodall EA, Greene ER, Jonsson E, Dong KC, Martin A. Structure and Function of the 26S Proteasome. Annu Rev Biochem. 2018; 87: 697-724.

[2] Li Y, Huang J, Zeng B, Yang D, Sun J, Yin X, Lu M, Qiu Z, Peng W, Xiang T, Li H, Ren G. PSMD2 regulates breast cancer cell proliferation and cell cycle progression by modulating p21 and p27 proteasomal degradation. Cancer Lett 2018; 430: 109-122.

[3] Nijhawan D, Zack TI, Ren Y, Strickland MR, Lamothe R, Schumacher SE, Tsherniak A, Besche HC, Rosenbluh J, Shehata S, Cowley GS, Weir BA, Goldberg AL, Mesirov JP, Root DE, Bhatia SN, Beroukhim R, Hahn WC. Cancer vulnerabilities unveiled by genomic loss. Cell 2012; 150(4): 842-854.

2. With the addition of comments on other E3 ligases, the Discussion is much improved. However, the majority of the Discussion is still a re-hashing of the Results section. It might be helpful to readers if you could place your findings in a broader context, perhaps by briefly considering future directions and new questions presented by your work.

**Reply:** According to the reviewer’s comment, we have revised the Discussion section and placed our findings in a broader context by briefly considering future directions of the research.
